# Reframing simulation as an implementation strategy: a call for conceptual integration across two scientific communities

**DOI:** 10.1186/s43058-026-01048-0

**Published:** 2026-07-28

**Authors:** Elizabeth Sanseau, Anne Adema, Marc Auerbach, Bethany M. Kwan

**Affiliations:** 1 ¹Division of Emergency Medicine, Department of Pediatrics, Children’s Hospital of Philadelphia, University of Pennsylvania Perelman School of Medicine, Philadelphia, PA USA; 2 ²Department of Pediatrics, Section of Emergency Medicine, University of Colorado Anschutz Medical Campus, Aurora, CO USA; 3 ³Yale School of Medicine, New Haven, Connecticut, USA; 4 ⁴Department of Emergency Medicine, University of Colorado Anschutz Medical Campus, Aurora, CO USA

**Keywords:** Simulation, Implementation strategy, ERIC taxonomy, Simulation-based medical education, Translational simulation, Systems testing, Quality improvement, Implementation science

## Abstract

**Background:**

Simulation is an established evidence-based tool in healthcare. It is widely used for medical education (simulation-based medical education [SBME]), quality improvement, capacity building, audit and feedback, and probing healthcare systems for latent safety threats. Implementation science (IS) currently subsumes these functions under broad taxonomy categories such as “conduct ongoing training” or “cyclical tests of change.” This framing fails to capture simulation’s distinct mechanisms and its unique potential to close the evidence-to-practice gap. We argue that the time has come for implementation scientists and the healthcare simulation community to come together around a shared question: does simulation, as it is already practiced in healthcare, meet the criteria for a discrete, named implementation strategy within taxonomies such as the Expert Recommendations for Implementing Change (ERIC)?

**Main body:**

Drawing on the ERIC definition of an implementation strategy as “methods or techniques used to enhance the adoption, implementation, and sustainability of a clinical program or practice,” we argue that simulation in healthcare already functions in this role across at least three distinct domains: developing individual and team competencies (SBME); probing healthcare systems for latent hazards and driving quality improvement; and operationalizing evidence-based interventions (EBIs) and clinical decision supports (CDS) under realistic conditions. We specify simulation using established IS frameworks—including the Consolidated Framework for Implementation Research (CFIR), the EPIS framework (Exploration, Preparation, Implementation, Sustainment), and the PRISM (Practical, Robust Implementation and Sustainability Model)—and propose a research agenda at the intersection of these two fields. High-reliability industries including aviation, astronautics, and nuclear power provide useful analogies, but the healthcare simulation literature has developed a robust and distinct evidence base in its own right, exemplified by translational simulation paradigms.

**Conclusion:**

Simulation is not simply a training technique. It is a multi-mechanism, multi-level strategy with proven applications in healthcare education, QI, and systems change. Formally recognizing simulation within IS strategy taxonomies would sharpen conceptual clarity, enable more precise evaluation, and open a productive dialogue between two scientific communities whose work is more convergent than current frameworks suggest.

**Table:** 

Text box 1. Contributions to the literature
• We argue that healthcare simulation—already used for education, QI, systems probing, and EBI adoption—meets the ERIC definition of a discrete implementation strategy and warrants formal recognition in IS taxonomies.
• We offer a full specification of simulation as an implementation strategy (actors, actions, targets, mechanisms, temporality) that distinguishes it from generic “training” strategies.
• We map simulation’s existing applications onto CFIR, EPIS, and PRISM, demonstrating substantive rather than surface-level alignment.
• We propose a research agenda and practical entry points for IS researchers and simulationists to collaborate, including guidance on study design and accessing simulation partnerships and resources.

## Background

Simulation in healthcare encompasses a spectrum of structured, experiential methods designed to replicate clinical tasks, team dynamics, or system behaviors in a safe environment. In general, simulationists function across hybrid contexts, including but not limited to in situ simulation within healthcare settings, in-person or virtual simulation center–based activities, and fully virtual simulation environments. For a comprehensive reference of simulation terminology, modalities, and related concepts, readers are referred to the Society for Simulation in Healthcare’s *Healthcare Simulation Dictionary* [1]. Its most widely recognized application is simulation-based medical education (SBME)—training clinicians in procedural skills, clinical decision-making, and teamwork [2]. The evidence base for SBME is well established: it improves individual competence, teamwork, knowledge retention, and—where studied—patient outcomes [2].

Yet SBME represents only part of what simulation does in healthcare. A parallel and growing body of literature documents simulation used explicitly for systems-level purposes: uncovering latent safety threats before new units open [3], informing multimillion-dollar facility decisions through human factors analysis [4], and providing a comprehensive framework for clinical systems testing from intake through implementation [5]. The translational simulation paradigm, developed by Brazil and colleagues, crystallizes this distinction by asking not “where?” but “why?”—prioritizing the functional intent of simulation (quality improvement and system change) over its setting or modality [6, 7]. Complementary work has documented the integration of simulation with QI infrastructures across healthcare settings.

Despite this breadth, implementation science (IS) has largely subsumed simulation under blunt taxonomy categories. The Expert Recommendations for Implementing Change (ERIC)—a landmark compilation of 73 discrete implementation strategies organized by expert consensus [8]—includes categories such as “conduct ongoing training,” “conduct educational outreach visits,” and “conduct cyclical small tests of change.” Simulation overlaps with all of these, yet is not named as a strategy in its own right. Dubrowski and Dubrowski identified this gap from the simulation side, arguing that IS methods are urgently needed to answer fundamental questions in simulation-based health professions education: why do some simulation programs succeed while others fail, and how do we shift from letting implementation happen to making it happen [9]? What has been missing is the reciprocal move: asking whether simulation itself, as practiced in healthcare, belongs in the IS strategy taxonomy.

We propose that it does—and that recognizing this would benefit both communities.

## Main text

### What is an implementation strategy, and does simulation qualify?

Proctor et al. define implementation strategies as “methods or techniques used to enhance the adoption, implementation, and sustainability of a clinical program or practice” [10]. Powell et al.’s ERIC taxonomy operationalizes this definition across 73 discrete strategies, organized under nine expert-derived clusters [8]. The ERIC authors explicitly anticipated future refinement: “Future phases of ERIC will focus on developing conceptually distinct categories of strategies” and extending the taxonomy as the field develops.

Simulation in healthcare meets the Proctor et al. definition across multiple domains. Consider three:

First, SBME as an implementation strategy for clinical guideline adoption. When an interprofessional team rehearses a new sepsis protocol using a high-fidelity simulation scenario, they are not merely learning—they are enacting the protocol, surfacing barriers to its use (e.g., order set navigation, communication failures), building shared mental models, and generating feedback that enables iterative refinement before the protocol goes live. This is not “training” in the generic sense; it is a structured, evidence-based mechanism for enhancing the adoption and fidelity of a specific clinical practice.

Second, simulation for systems integration and latent hazard detection. Colman and colleagues describe simulation-based clinical systems testing applied prospectively—before a new space, workflow, or technology goes live—to identify failure modes that would otherwise only emerge in real patient care [5]. Reid and colleagues applied this approach specifically in pediatric emergency medicine [11]. Dubé and colleagues used human factors simulation to inform a major healthcare facility decision [4]. These are not educational activities; they are prospective systems probes designed to enhance the implementation and sustainability of new operational environments.

Third, simulation for evidence-based intervention (EBI) operationalization. Teams can use simulation to practice applying clinical decision support (CDS) tools, checklists, and care pathways under realistic conditions—identifying not only knowledge gaps but workflow incompatibilities, usability barriers, and team coordination failures. This iterative rehearsal-and-debrief cycle directly addresses the “know-do gap” that IS was founded to close.

Across all three domains, simulation functions as a method used to enhance adoption, implementation, and sustainability of clinical programs or practices. It meets the definitional threshold.

### What makes simulation distinct from existing ERIC strategies?

Simulation overlaps with multiple ERIC strategies simultaneously—it can be a form of ongoing training, a cyclical test of change, an audit-and-feedback mechanism, a facilitation method, and a coalition-building exercise all within a single scenario. But this is precisely what makes it distinct rather than redundant: no single ERIC strategy, nor any obvious bundle of strategies, captures what simulation does when it functions as a prospective systems probe.

The distinctiveness lies in simulation’s mechanisms. Unlike didactic education, simulation creates a psychologically safe environment for rehearsal under realistic conditions—generating behavioral and systems data that other strategies cannot. Unlike standard audit-and-feedback, simulation surfaces latent hazards before they produce adverse events. Unlike traditional facilitation, simulation can engage the full interprofessional team in a shared behavioral experience that builds common ground, exposes hidden assumptions, and enables real-time adaptation.

Specifying simulation as a named strategy—with actors, actions, targets, mechanisms, temporality, and attributes defined—would not replace existing ERIC categories but would add conceptual precision. Table 1 provides this specification, drawing on established IS frameworks for strategy specification.

**Table 1:** Specification of simulation as an implementation strategy

Element	Description / Considerations
Strategy name	Simulation
Definition	Use of structured, experiential methods that replicate real clinical tasks, workflows, or system dynamics—via physical, digital, or hybrid modalities—enabling rehearsal, barrier identification, adaptation, and feedback in a safe environment.
Actors	Simulation facilitators, clinicians, interprofessional teams, system engineers, administrators, organizational leaders.
Actions	Co-designing scenarios; conducting simulation events; facilitating debriefings; probing latent system hazards; adapting workflows; iteratively refining evidence-based interventions or clinical decision supports; stress-testing new processes before go-live.
Targets / Levels	Individuals (knowledge, skills, self-efficacy); Teams (communication, coordination, shared mental models); Microsystem (unit workflows, local culture); Mesosystem (inter-unit transitions); Macrosystem (organization-wide norms, safety culture).
Mechanisms	Knowledge translation; barrier detection; role clarity; safe experimentation; feedback loops; culture change; normalization of new practices.
Outcomes	Implementation outcomes (acceptability, feasibility, fidelity, adoption, sustainability); Service/system outcomes (safety event rates, process adherence, teamwork climate); Clinical outcomes as downstream metrics.
Temporality	Applicable across Exploration, Preparation, Implementation, and Sustainment phases (EPIS framework).
Distinguishing attributes	In situ simulation maximizes contextual fidelity and system probing; interprofessional simulation emphasizes multi-disciplinary team dynamics; translational simulation explicitly foregrounds healthcare quality improvement as the primary goal.

### Simulation aligns substantively with IS frameworks

Simulation does not merely overlap with IS frameworks—it engages them at the level of mechanism. Within the Consolidated Framework for Implementation Research (CFIR) [12], well-designed simulation can help surface inner-setting determinants (culture, communication patterns, workflow barriers, readiness) that structured interviews often cannot, because simulation makes tacit behaviors observable. Simulation may reveal individual-level determinants (clinician beliefs, confidence, skill gaps) and can test the adaptability and compatibility of EBIs under realistic operational pressure—engaging the intervention characteristics domain directly.

Within the EPIS (Exploration, Preparation Implementation and Sustainment) model [13], simulation is applicable across all four phases: scenario-based trials during Exploration to assess fit; rehearsal and adaptation of new workflows during Preparation; just-in-time simulation to support Implementation; and periodic normalization drills to promote Sustainment. The PRISM (Practical, Robust Implementation and Sustainability Model) [14] similarly maps well: simulation addresses the contextual factors, stakeholder engagement, and sustainability determinants that PRISM identifies as critical.

These alignments are substantive rather than surface-level. They suggest that simulation, when explicitly conceptualized and evaluated as an implementation strategy, would be tractable to the same rigorous evaluation frameworks IS applies to other strategies.

### Precedent from high-reliability industries

High-reliability industries provide useful, if imperfect, precedent. In aviation, simulator-based competency assessment is mandated by regulation—not recommended—reflecting a societal commitment to safety that is independent of return-on-investment calculations [15]. Military training has long used simulation as a systems-readiness mechanism, not merely a skills-training tool [16].

These analogies do not prescribe healthcare’s approach. Healthcare simulation operates in different institutional, ethical, and organizational contexts. But they affirm that the concept of simulation-as-systems-probe is well validated in complex, high-stakes domains—and that healthcare has both the evidence base and the professional infrastructure to operationalize it in contextually appropriate ways.

### Opportunities, challenges, and a research agenda

Formally recognizing simulation as an IS strategy would open several productive research directions. Studies are needed that elucidate the mechanisms through which simulation achieves implementation outcomes—distinguishing, for example, between skill acquisition pathways in SBME and process-redesign pathways in systems probes. Comparative effectiveness research could examine which simulation modalities (in situ, center-based, telesimulation, hybrid) work best for which implementation goals, at which organizational levels, and under what contextual conditions.

A meaningful limitation is simulation’s historical concentration in acute care, hospital-based environments. Telesimulation and low-fidelity in situ approaches—tabletop scenarios, structured role-play, manikin-free walkthroughs—show promise for community health, primary care, and resource-limited settings [17], and future work should explicitly test and report simulation-based IS strategies in these contexts.

Resource intensity remains a real constraint. Organizing simulation requires coordination of participants, facilitators, and equipment. However, the case for simulation as a systems-safety strategy need not rest on return-on-investment alone. A growing economics literature documents feasible models, including malpractice carrier partnerships [18]. The Improving Pediatric Acute Care through Simulation (ImPACTS) consortium demonstrates how multi-site simulation research can be conducted at scale [19].

For IS researchers seeking to incorporate simulation: collaborators can be identified through the Society for Simulation in Healthcare (SSH) and the International Nursing Association for Clinical Simulation and Learning (INACSL). SSH-accredited programs now extend well beyond academic medical centers to community hospitals and pediatric facilities. Freely accessible, peer-reviewed simulation scenarios are available through MedEdPORTAL and the Centre for Medical Simulation Free Open Access Materials and can be adapted for implementation purposes.

The most important research question is definitional: under what conditions does simulation function as an implementation strategy rather than as an educational curriculum being implemented? Clarifying this distinction—following the lead of Dubrowski and Dubrowski’s IS primer for simulation programs [20], and of the broader ERIC specification work [8, 10]—would transform simulation from a locally practiced art into a coherent, evaluable component of IS strategy libraries.

## Conclusions

Healthcare simulation is not simply a training technique. It is a multi-mechanism, multi-level approach with an established evidence base spanning education, quality improvement, human factors, and systems change—and it meets the ERIC definition of an implementation strategy. Yet it remains insufficiently characterized within implementation science strategy taxonomies, subsumed under broad categories that obscure its distinctive mechanisms and unique capacity for prospective systems probing, as well as its ability to test, implement, and sustain adoption of EBIs and CDS.

**Fig. 1:**
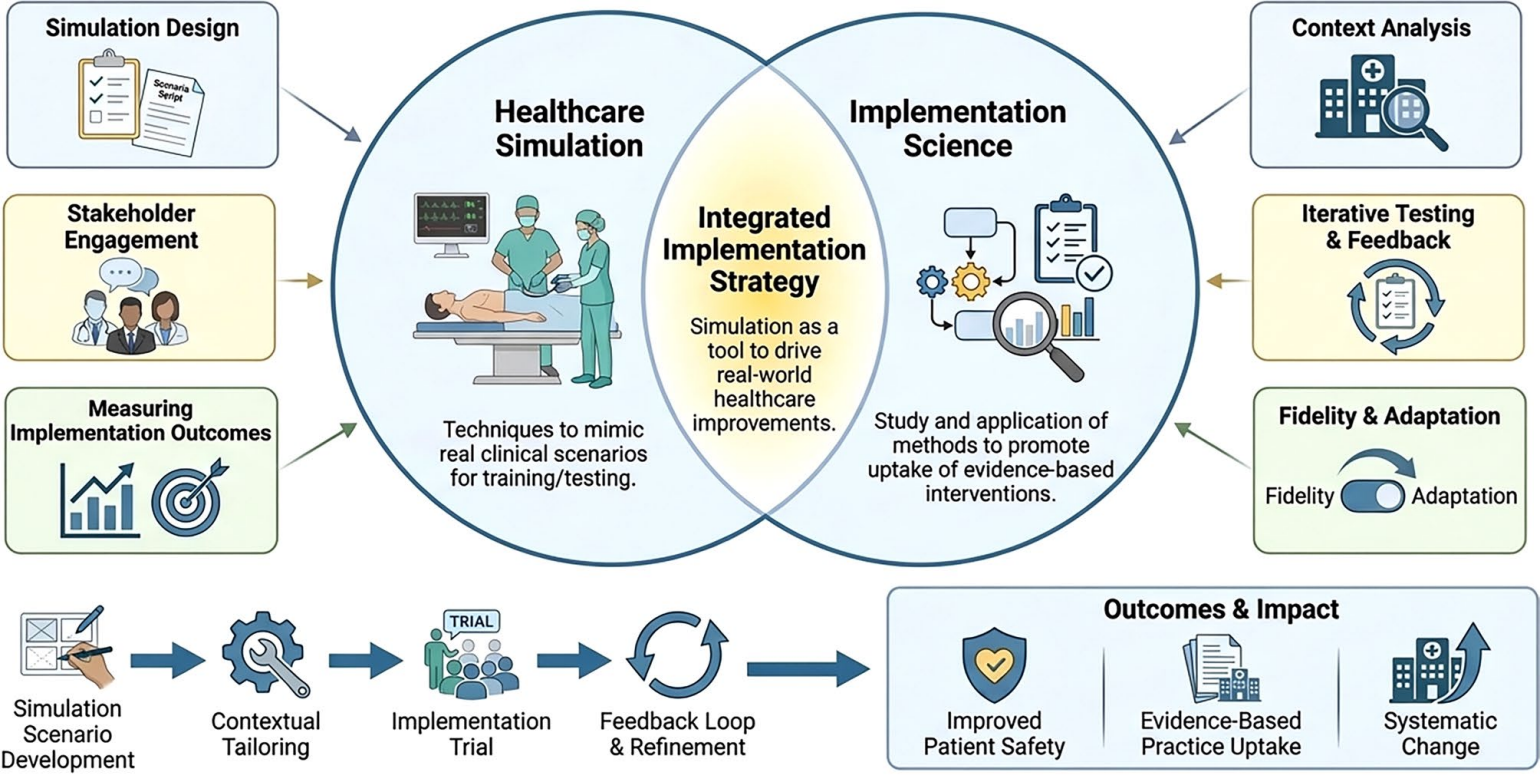
Reframing healthcare simulation as a distinct implementation strategy: integrating simulation with implementation science. Legend: Conceptual framework illustrating the integration of healthcare simulation and implementation science as an “Integrated Implementation Strategy” to drive real-world healthcare improvement. The left circle represents healthcare simulation, encompassing techniques used to mimic clinical scenarios for training, systems testing, and identification of latent safety threats. The right circle represents implementation science, focused on the systematic study and application of methods to promote the uptake, integration, and sustainment of evidence-based interventions in routine practice. The overlapping center highlights simulation’s proposed role as a distinct implementation strategy that can actively facilitate implementation processes rather than serving solely as an educational modality. Surrounding domains depict core components informing this integrated approach, including simulation design, stakeholder engagement, measurement of implementation outcomes, context analysis, iterative testing and feedback, and balancing fidelity with contextual adaptation. The bottom pathway demonstrates a cyclical implementation process beginning with simulation scenario development and contextual tailoring, followed by implementation trials, iterative feedback and refinement, and progression toward downstream outcomes. Anticipated outcomes include improved patient safety, greater uptake of evidence-based practices, and broader systems-level change. This framework proposes that simulation possesses unique mechanisms capable of supporting implementation across multiple phases of implementation, including exploration, preparation, implementation, and sustainment, and should therefore be conceptualized and evaluated as a distinct implementation strategy within healthcare systems

We call on implementation scientists and healthcare simulationists to begin the work that the ERIC authors anticipated: developing conceptually distinct, rigorously specified strategy categories that reflect how the field actually operates. Simulation is a natural and overdue candidate for inclusion. Formalizing its role in IS taxonomies would sharpen evaluation, enable cross-study comparison, and ultimately help close the evidence-to-practice gap that both communities exist to address. See Fig. 1*.*

The two communities have been doing related work in parallel for long enough. The evidence, the frameworks, and the professional infrastructure are all in place. What remains is the conversation.

## Data Availability

No datasets were generated or analysed during the current study.

## Abbreviations

CFIR: Consolidated framework for implementation research
CDS: Clinical decision support
EBI: Evidence-based intervention
EPIS: Exploration preparation implementation sustainment framework
ERIC: Expert recommendations for implementing change
IS: Implementation science
PRISM: Practical robust implementation and sustainability model
QI: Quality improvement
SBME: Simulation-based medical education
